# Data on cephalexin removal using powdered activated carbon (PPAC) derived from pomegranate peel

**DOI:** 10.1016/j.dib.2018.08.204

**Published:** 2018-09-07

**Authors:** Yousef Rashtbari, Sadegh Hazrati, Shirin Afshin, Mehdi Fazlzadeh, Mehdi Vosoughi

**Affiliations:** a Student Research Committee, Department of Environmental Health Engineering, School of Public Health, Ardabil University of Medical Sciences, Ardabil, Iran; b Department of Environmental Health Engineering, School of Public Health, Ardabil University of Medical Sciences, Ardabil, Iran; c Social Determinants of Health Research Center, Ardabil University of Medical Sciences Ardabil, Iran; d Department of Environmental Health Engineering, School of Public Health, Tehran University of Medical Sciences, Tehran, Iran

**Keywords:** Cephalexin, Powdered activated carbon, Pomegranate peel

## Abstract

Cephalexin is extensively used as an antibiotic for treatment a number of bacterial infections. The data of possible adsorption mechanism and isotherm of Cephalexin on the synthesized adsorbent are depicted in this data article. The data obtained showed that the adsorption trend follows the pseudo-second order kinetic model and that the Langmuir isotherm was suitable for correlation of equilibrium data with the maximum adsorption capacity of 48.78 mg/g. Considering the findings data, powdered activated carbon derived from pomegranate peel as available and a cheap adsorbent, could be considered as promising adsorbent for Cephalexin and probably similar organic pollutants removal from aqueous solutions.

**Specifications table**

**Table:** 

Subject area	*Environmental Engineering*
More specific subject area	1. *Industrial effluent treatment* 2. *Wastewater technology*
Type of data	*Tables, Figures, Images*
How data was acquired	1. *Pomegranate peel extract was used to synthesize zinc oxide nanoparticles.* 2. *Batch experiments were performed to collect the data of the influence of contact time and pH on cephalexin removal.* 3. *scanning electron microscopy (SEM) images using a Philips-FEI XL30, Philips X’PertPro instrument (Netherlands), pH meter (Sense Ion 378, Hack), double beam spectrophotometer (Model lambda 25- Perkin Elmer Company) and Eppendorf versatile 5810 series centrifuge were used.* 4. *The obtained data were analyzed using appropriate equations and isotherm and kinetic models.*
Data format	*Analyzed*
Experimental factors	1. *The adsorbent of PPAC was prepared from waste of Pomegranate Peel that has been collected from juice shops.*
	*Pomegranate Peel was sieved through a 60 mesh screen and then it was soaked with phosphoric acid (85 wt. % H*_*3*_*PO*_*4*_*) in the ratio of 1:1 (w/w) for 48 h*
	1. *The adsorbent of PPAC was heated in the furnace at 800 °C for 2 h to burn organic contents, then dried in an oven for 2 h at 110 °C.* 2. *Data of PPAC were obtained for cephalexin removal from aqueous solution*
Experimental features	*Preparation of powdered activated carbon from pomegranate peel and its performance for the adsorption of cephalexin from aqueous solutions. Characterization data of powdered activated carbon obtained from SEM and XRD analyses are given.*
Data source location	*Ardabil city, Ardabil province, Iran*
Data accessibility	*Data are available in article*
Related research article	*Please add author names, title and publication details/status of the most relevant research article here, if available*

**Value of the data**

• This data offers a simple and environmentally friendly method for preparation of activated carbon from pomegranate peel.
• This data will be useful for the water scientific community to design an adsorption column with adsorbent of PPAC as medium for the removal of cephalexin-containing waters or wastewaters.
• Characterization data for PPAC derived from pomegranate peel as the newly synthesized adsorbent are given.
• The data of isotherms and kinetics will be informative and useful for predicting and modeling of the adsorption and mechanism of cephalexin removal from aqueous solutions by PPAC.

## 1 **Data**

**Fig. 1:**
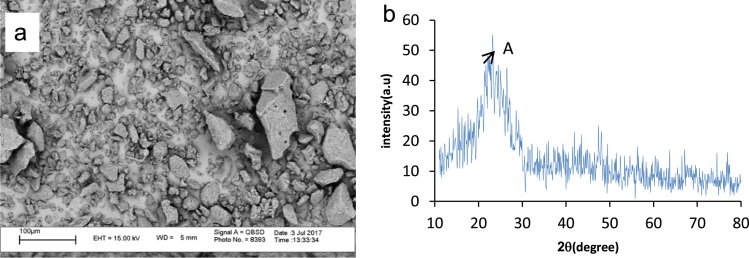
(a) SEM micrograph of PPAC; and (b) XRD patterns of PPAC.

**Fig. 2:**
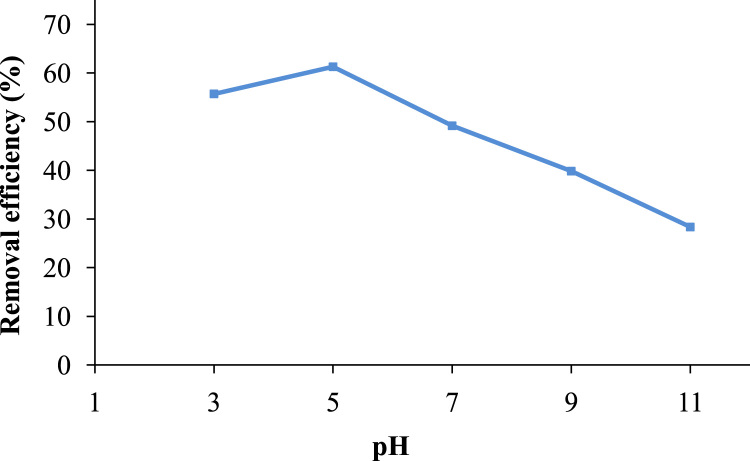
The effect of solution pH on the CEX removal by PPAC (initial concentration =50 mg/L, optimum dose=0.75 g/L, and contact time = 30 min, shaking speed = 200 rpm at room temperature).

**Fig. 3:**
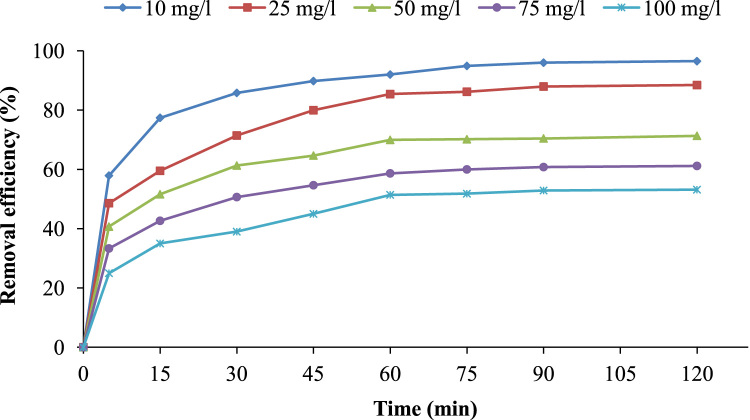
Effect of contact time on cephalexin adsorption by PPAC (adsorbent dose =1.25 g/L, pH=5, shaking speed = 200 rpm at room temperature).

The pomegranate peel was collected from juice shops in Ardabil city in Iran, for the preparation of *powdered activated carbon*. Scanning electron microscopy (SEM), and Philips X’Pert Pro instrument (the Netherlands) were used to obtain particle sizes and XRD patterns of the PPAC, respectively. The obtained data are shown in Fig. 1(a) and (b). The effects of solution pH and contact time on removal efficiency are presented in Fig. 2, Fig. 3, respectively. The equations of the studied isotherm and kinetic models are presented in Table 1. The kinetic and isotherm data are also shown in Table 2, Table 3, respectively.

**Table 1:** The equations of the studied isotherm and kinetic models [6], [7], [8], [9], [10].

**Model types**	**Name**	**Equation**
**Isotherm models**	Langmuir	1/q_e_=1/(q_m_K_1_C_e_)+(1/q_m_)
	Freundlich	logq_e_=log k_f_ +(1/n)logC_e_
**Kinetic models**	Pseudo first order	Log(q_e_-q_t_)=logq_e_-(k_1_/2.303)t
	Pseudo second order	t/q_e_=1/(k_2_$q_{e}^{2}$)+1(1/q_e_)t

**Table 2:** Kinetic parameters of the pseudo-first order and pseudo-second order models for the removal of CEX by PPAC [11].

**C_0_ (mg/L)**	**Pseudo-first-order**				**Pseudo-second-order**			
	**qe,exp (mg/g)**	**k_1_ (min^-1^)**	**qe,cal (mg/g)**	**R**^**2**^	**k_2_ (g/mg min)**	**qe,cal (mg/g)**	**R^2^**	**h (mg/g min)**
**10**	8	0.025	3.51	0.8644	0.04	7.88	0.9988	2.508
**25**	17.8	0.041	11.84	0.979	0.01	18.34	0.9961	3.632
**50**	28.8	0.037	15.57	0.9404	0.008	29.32	0.9977	7.336
**75**	36.9	0.042	23.35	0.9796	0.006	37.73	0.9973	8.733
**100**	42.65	0.048	37.26	0.9793	0.003	44.64	0.9917	6.548

**Table 3:** Isotherm parameters for adsorption of CEX onto PPAC.

**Langmuir**				**Freundlich**		
**R_L_**	R^2^	q_m_(mg/g)	K_L_(L/mg)	R^2^	K_F_(mg/g)	n
**0.18**	0.9869	48.78	0.181	0.9774	8.87	2.47

## 2 Experimental design, materials and methods

### 2.1 Materials

All chemicals materials were purchased from Merck. Cephalexin (CEX) with purity 97% (C_16_H_17_N_3_O_4_S; MW=347.39 g/mol) was obtained from Sina Daru Co in Iran. The stock of synthetic CEX (1000 mg/L) was made by dissolving the required amount in deionized water and kept in a glass container at 4 °C in darkness [1], [2], [3].

### 2.2 Preparation of powdered activated carbon (PPAC)

Pomegranate Peel was collected from juice shops in Ardabil city in Iran and then washed several times with distilled water to remove dust and impurities and then dried in an oven for 2 h at 100 °C [4]. The well-grounded material was sieved through a 60 mesh screen and then it was soaked for 48 h in the ratio of 1:1 (w/w) with phosphoric acid (85 wt. % H_3_PO_4_). The dried material was placed in a cylindrical steel reactor in furnace (5 °C/min) for 2 h at 800 °C. After cooling samples of pomegranate peel active carbon (PPAC) washed several times with distilled water to reach a neutral pH and was dried in an oven for 2 h at 110 °C. Finally, PPAC was stored in a desiccator for further use [5].

### 2.3 Determination of Cephalexin content and adsorption–desorption experiments

The effects of contact time, initial CEX concentration, pH, adsorbent dosage, temperature, and competing ions on the CEX adsorption efficiency were carried out in a batch manner in 100 ml conical flasks at 21 ± 1 °C. Synthetic CEX solution with initial concentration of 50 mg/L was prepared from a 1000 mg/L stock solution of Cephalexin. pH solution for each experiment was adjusted by using 0.1 M NaOH or H_2_SO_4_. Then, determined amounts of absorbent weights were added to the Erlenmeyer flasks. Thereafter, it was agitated at 200 rpm until predetermined contact time. After desired contact time, the samples were centrifuged and filtered using a Whattman paper (0.2 µm) [12] and finally the filtered sample was analyzed by double beam spectrophotometer (Model lambda 25-Perkin Elmer Company) at the maximum absorption wavelength of 261 nm to determine the residual cephalexin concentration [1].

The CEX removal efficiency (%) and the equilibrium adsorption capacity (qe, mg/g) was determined using Eqs. (1), (2), respectively [13], [14], [15], [16]:

(1) $$R\%=\left[\frac{(C_{o}-\mathrm{Ce})}{C_{o}}\right]\times 100$$

(2) $$qe=\frac{\left[{(C}_{o}-\mathrm{Ce})\times V\right]}{m}$$

Where C_0_ and C_e_ are the initial and equilibrium concentration of the CEX (mg/L) respectively, V is the volume of the CEX solution (L) and m is the mass of adsorbent used (g).
