# Supplementary material for: Data on cephalexin removal using powdered activated carbon (PPAC) derived from pomegranate peel
Source: Data Brief. 2018 Sep 7;20:1434–9. doi: 10.1016/j.dib.2018.08.204 (PMC6148726; doi:10.1016/j.dib.2018.08.204)
Supplement: Supplementary file 1 — Supplementary material [file mmc1.docx]

Conflict of Interest

The authors of this article declare that they have no conflict of interests.

Best regards

Mehdi fazlzadeh
